# Multi-host disease management: the why and the how to include wildlife

**DOI:** 10.1186/s12917-019-2030-6

**Published:** 2019-08-14

**Authors:** Julien Portier, Marie-Pierre Ryser-Degiorgis, Mike R. Hutchings, Elodie Monchâtre-Leroy, Céline Richomme, Sylvain Larrat, Wim H. M. van der Poel, Morgane Dominguez, Annick Linden, Patricia Tavares Santos, Eva Warns-Petit, Jean-Yves Chollet, Lisa Cavalerie, Claude Grandmontagne, Mariana Boadella, Etienne Bonbon, Marc Artois

**Affiliations:** 1 Sciences&Faunes, Créteil, France; 2 Centre for Fish and Wildlife Health (FIWI), Vetsuisse-Faculty, University of Bern, Bern, Switzerland; 3 Scotland’s Rural College (SRUC), Edinburgh, EH9 3JG UK; 4 ANSES, Nancy Laboratory for Rabies and Wildlife, Malzéville, France; 5 FaunaVetService, Pluvigner, France; 6 Wageningen BioVeterinary Research, Houtribweg 39, 8221 RA Lelystad, The Netherlands; 7 Status Department, OIE, Paris, France; 8 Surveillance Network of Wildlife Diseases, Department of Infectious Diseases, Faculty of Veterinary Medecine, University of Liege, Liege, Belgium; 9 Direção Geral de Alimentação e Veterinária, Lisbon, Portugal; 10 Direction Départementale de la Cohésion Sociale et de la Protection des Populations d’Ille-et-Vilaine, Rennes, France; 11 Direction de la Recherche et de l’Expertise, Office National de la Chasse et de la Faune Sauvage, BP 20, 78612 Le Perray-en-Yvelines, France; 12 Animal Health Office, General Directorate for Food, French Ministry of Agriculture, Paris, France; 13 Association Animal Société Aliment, Maisons-Alfort, France; 14 Sabiotec, Camino de Moledores s.n., Ed. Polivalente UCLM, Madrid, Spain; 15 Commission des normes sanitaires pour les animaux terrestres, OIE, Paris, France; 16 LISAE, Lagney, France

**Keywords:** Emerging infectious diseases, Zoonosis, Proportionate management, Risk assessment, Europe, Coordination, Decision-making framework, Wildlife, Integrated management, Policy making

## Abstract

In recent years, outbreaks caused by multi-host pathogens (MHP) have posed a serious challenge to public and animal health authorities. The frequent implication of wildlife in such disease systems and a lack of guidelines for mitigating these diseases within wild animal populations partially explain why the outbreaks are particularly challenging. To face these challenges, the French Ministry of Agriculture launched a multi-disciplinary group of experts that set out to discuss the main wildlife specific concepts in the management of MHP disease outbreaks and how to integrate wildlife in the disease management process.

This position paper structures the primary specific concepts of wildlife disease management, as identified by the working group. It is designed to lay out these concepts for a wide audience of public and/or animal health officers who are not necessarily familiar with wildlife diseases. The group’s discussions generated a possible roadmap for the management of MHP diseases. This roadmap is presented as a cycle for which the main successive step are: step 1-descriptive studies and monitoring; step 2-risk assessment; step 3-management goals; step 4-management actions and step 5-assessment of the management plan. In order to help choose the most adapted management actions for all involved epidemiological units, we integrated a decision-making framework (presented as a spreadsheet). This tool and the corresponding guidelines for disease management are designed to be used by public and health authorities when facing MHP disease outbreaks. These proposals are meant as an initial step towards a harmonized transboundary outbreak response framework that integrates current scientific understanding adapted to practical intervention.

**Electronic supplementary material:**

The online version of this article (10.1186/s12917-019-2030-6) contains supplementary material, which is available to authorized users.

## Background

Diseases caused by multi-host pathogens (MHP) that affect at least one free-living wild animal species may deeply challenge veterinary services in their missions to manage threats for human health, the animal trade economy and biodiversity conservation [1]. In Europe, outbreaks of animal tuberculosis (TB), African swine fever (ASF), or rabies illustrate the difficulty of maintaining an international status of freedom when a pathogen that can be transmitted to humans or livestock circulates in a wild animal population [1]. TB eradication in livestock is notoriously challenging in the presence of wild maintenance hosts such as badgers (UK), wild boars (Spain) or [2] white-tailed deer (USA) [3].

Wildlife is recognized as a source of many emerging infectious diseases (EIDs) in humans as well as in livestock [4–6]. This emergence largely results from increased interactions between humans, livestock and wildlife [7, 8]. Thus, in addition to the wildlife health challenges that Europe has been facing for several decades (rabies, TB…), emerging infectious events implicating wildlife have become another major preoccupation for animal health authorities. Examples of particular concerns include ASF, chronic wasting disease and West Nile virus infection.

Successful attempts at eradicating or severely reducing the occurrence of a disease implicating wildlife were achieved only when management plans were implemented specifically for wildlife, as was the case with the massive vaccination campaign against fox rabies in Western Europe. However, while accepted operating procedures are often available for human and livestock disease control, they are uncommon for wildlife, which is largely related to the difficulties associated with the health assessment of, and disease control in, wildlife populations. Current advances in ecological understanding along with improvements in disease surveillance and control tools mean that we are more likely to be successful at detecting and controlling disease outbreaks. This in turn makes it a good time to consider a common approach to better integrate wildlife in the control of diseases caused by MHPs. The European Union (EU) has endorsed this necessity: the new Regulation on transmissible animal diseases [9] states that “Special rules should […] be laid down, where necessary, for measures to control and eradicate diseases in wild animals”.

To efficiently tackle diseases implicating wildlife, public health officers should initially be familiarized with the specific concepts of disease management in wildlife. Following this suggestion and with the financial support of the French Ministry of Agriculture, a group of European experts gathered to prepare a state-of-the-art accounting of the current methods and tools used to manage diseases and infections that are notifiable to the World Organisation of Animal Health (OIE) when the involved pathogens are harboured by wildlife. The members of this working group were selected by the ASA organization (Animal Société Aliment) to encompass a range of professional expertise including game and wildlife managers, veterinary pathologists, public and veterinary public health officers, microbiologists, ecologists, and representatives of the French Ministries for Agriculture and for Ecology. All of these experts are co-authors of this paper.

This position paper structures the basic concepts of wildlife disease management for a wide audience of public and animal health officers who are not necessarily familiar with wildlife diseases. From the concepts discussed in this article, a basis emerged for operational guidelines which could be used by public and animal health authorities when facing MHP disease outbreaks (i.e., how to include wildlife?). These guidelines are meant to ensure that the concepts of wildlife disease management are not only respected but also integrated into the wider process of managing MHP diseases. This global process is illustrated as a roadmap for the management of MHP diseases, integrating a preliminary decision-making framework for choosing management actions across all susceptible populations. This proposal is meant as an initial step towards a common global outbreak response process that integrates current scientific understanding and is tailored for practical intervention.

The professional background of the experts is primarily focused on European wildlife health. As a consequence, although the concepts we develop are more global, the operational tools we propose are largely adapted to the European context.

## Main text

### Difficulties specific to the management of MHPs involving wildlife

MHPs can affect three types of epidemiological units, i.e., of vertebrate host populations [10, 11] as follows: (1) humans (zoonotic diseases), (2) captive animals, i.e., animals under human supervision, whether wild or domestic (such as domestic and zoo animals, farmed game or exotic pets), and (3) wildlife, understood here as free-living wild animal populations. The environment constitutes a fourth unit that is relevant to management when it enables pathogen persistence and/or transmission independently of a vertebrate host, as is the case for vector-borne diseases. From now on, we designate these four epidemiological units as “units*”* for the purpose of simplification. All four units can contribute to pathogen transmission and have to be considered for an efficient disease control effort.

For any population, the success of management actions relies on the ability to encompass a sufficient proportion of individuals within a short time-frame to affect the turnover of the infection (one-shot concept). This is much more difficult to achieve for wildlife than for animals living under human supervision: free-living wild animals are not restrained; their populations often cannot be quantified with useful accuracy; they interact in an uncontrollable manner with multiple other wild species and with an unfenced environment; and they are difficult to handle or even simply to approach on a short distance. As a consequence, disease management actions that may be successful in livestock cannot simply be transposed to wildlife.

Management actions are more complex and take longer to be implemented for wildlife. As an example, a vaccination plan on a wild species requires testing vaccines on that species (vaccines have usually been tested only in domestic species), developing and testing an administration form (for example oral baits) adequate for the target species, both in the laboratory and in the field), and ultimately planning a campaign of several months or years to vaccinate a sufficient proportion of the population. Such vaccination campaigns might also expose non-target species (especially with oral baits) to the vaccine, which can raise sanitary concerns for them [12]. For captive animals, when a vaccine is available, it is often presented as an authorized injectable formula which can be administered to all target animals within a relatively short period of time.

Some disease management actions such as culling are not as effective in wildlife as they have proven to be for captive animals; these actions have even been shown to be counter-productive in a number of situations [13]. For captive animals, a farm can be emptied of its stock and sanitized before introducing new individuals several weeks later. In the natural environment, total culling of a wild species population is rarely possible, especially within a short time-frame (a few weeks), and sanitation of the environment is generally not possible.

The management of diseases in wildlife is not only more complicated to implement, it is also subject to increased uncertainty regarding its expected efficiency and impacts because it is typically based on a lower level of knowledge than for captive animals, notably on population dynamics and host-pathogen interactions. Population size and structure (demographics) are often difficult to evaluate, although these are key parameters to evaluating transmission rates and setting up efficient management actions. The sanitary status of the animal population and the nature of its interactions with the pathogen (previous contacts between pathogen and hosts) also influence on transmission scenarios and ultimately the success of management plans. Wild animal populations may harbour a pathogen at a low prevalence but contribute to maintaining it locally, as is the case for classical swine fever (CSF) in wild boar (*Sus scrofa*) [14] or TB in red deer (*Cervus elaphus*) [15]. Furthermore, in such a scenario the detection of infections and the assessment of pathogen prevalence within a spatial unit, and the detection of trends over time – before, during and after the implementation of management actions – remain particularly challenging [16].

Due to the uncertainties associated with monitoring and managing a disease in wildlife, the consequences of management actions (beneficial or detrimental) are difficult to foresee and study. In the case of culling campaigns, immigration of new individuals and dispersion of infected individuals are difficult to predict and to prevent. Epidemiological and ecological investigations have to be planned carefully and conducted continuously to reduce the level of uncertainty all along the course of management actions.

In the event of a MHP disease outbreak, authorities often face contradictory positions and demands from stakeholders and lobbies. The complexity and diversity of MHP disease systems inevitably result in varying perceptions among them according to their own priorities. Farmers and other professional bodies from the private sector have sometimes been imposed extremely harsh measures for disease control on their own livestock for transboundary animal diseases (TADs), such as highly pathogenic avian influenza (HPAI), TB, ASF and CSF, with their on-farm detection resulting in total culling. These stakeholders may consider wildlife as a threat and call for more invasive management actions on this unit, which are in fact not necessarily efficient, nor safe, and sometimes not even possible for reasons discussed above. Furthermore, many wildlife species are protected (or/and emblematic), and disease management operations on these species may be illegal or at least meet the opposition of other interest groups, the general public, environmental NGO’s etc., as it is the case for a brucellosis outbreak (*Brucella melitensis*) in Alpine ibex (*Capra ibex ibex*) since 2012 [17].

In and of itself, the representation of wild animals may generate significantly different perceptions among environmental NGO’s, hunters, farmers and the general public - with several wildlife species being iconic for some and pests for others. The drivers of these varying perceptions depend not only on the hosts involved but also on pathogen virulence as well as on socio-cultural, economic and political factors. Such conflicts can also feed on the uncertainties regarding the epidemiological roles played by wildlife in such disease systems.

These specificities show that wildlife diseases cannot be monitored and managed in the same way and within the same time-frame as diseases of captive animals. This implies that the design of disease management plans should involve all of the disciplines relevant to each unit, including wildlife specialists who are competent in taking into account the specificities of wildlife (wildlife diseases, but also wildlife ecology and management, and social sciences). Namely, the following professionals should be included in the disease management process: medical doctors and public health officers in case of zoonotic disease, veterinarians knowledgeable on the concerned animal species, domestic and wild; ecologists (e.g., experts in population dynamics and ethologists), wildlife managers, and entomologists in the case of vector-borne diseases. Importantly, the role of wildlife managers in efficiently implementing the measures that are taken to limit an outbreak should be recognized and communicated, both at the national and European levels. Strategies should be developed according to a common framework to enable a coordinated response that accounts for all the relevant units and their specificities, including their interactions with the pathogen and with the other units.

In summary, to improve the outcome of managing MHP systems implicating wildlife, it is essential to (1) consider the wildlife unit for management actions, (2) take into account the specificities of the disease system in the wildlife unit and of disease management in wildlife and (3) integrate the management of the wildlife unit within the process of the wider management plan (integrating all other susceptible units).

### How to integrate wildlife specificities in the management of multi-host diseases: a logical process for decision making, adaptable to all situations

In the case of OIE notifiable diseases occurring in captive animals or humans, contingency plans often exist at national levels. These are powerful tools: they are designed in advance, schedule pre-defined actions to mitigate the disease and allow a swift official response to an outbreak. Whenever possible, we suggest such contingency plans to additionally include guidelines for pathogen control in wildlife if existing data suggest that wildlife may play an important role in the epidemiology of these diseases.

Such contingency plans are only possible to implement when the danger is identified and predictable. For all other MHP events, instead of pre-defined management actions, we suggest a common process for selecting, implementing and monitoring these actions. We propose a roadmap that consists in a succession of logical and transparent steps to cover the management process from the outbreak detection to the end of the disease management actions.

Preparing this roadmap during “peace” times should allow reaching a consensus among all stakeholders, at a time when there is still room for discussion and before emotions overshadow rational argumentation. Such a roadmap would not only contribute to reducing conflict risks, it would also enable quicker responses to disease events (reduced time spent agreeing on the process).

**Fig. 1:**
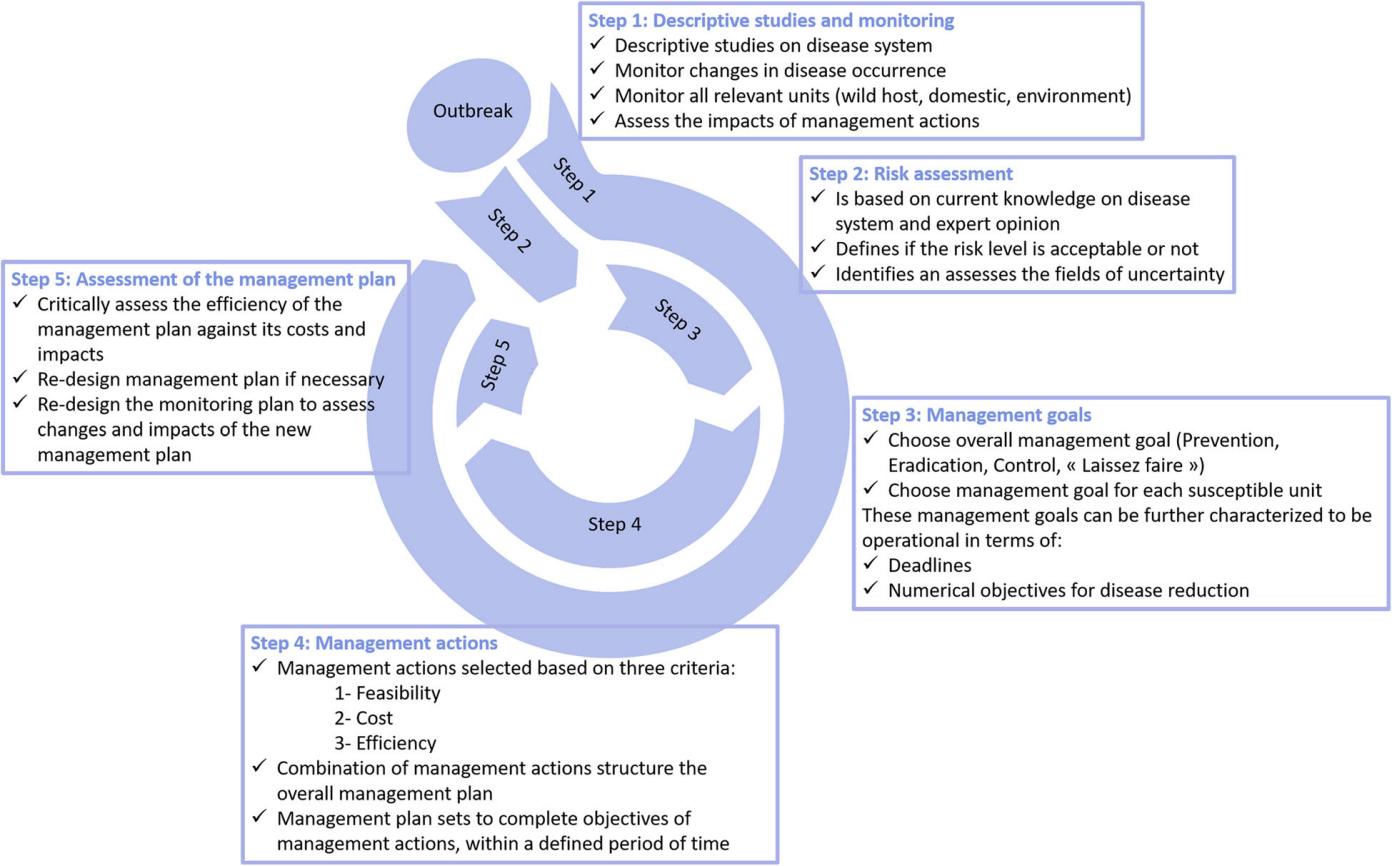
Management cycle for diseases implicating wildlife. Legend: Figure illustrating the roadmap designed for the management of diseases implicating wildlife. Step 1 -Descriptive studies and monitoring- is considered as ongoing for the whole duration of the outbreak (outer circle on the figure). Then the roadmap is constructed on 4 consecutive steps: (2) Risk assessment, (3) Management goals, (4) Management actions and (5) Assessment which are to be repeated as long as the outbreak persists (inner circle)

Based on the concepts presented here and in the following paragraphs, Fig. 1 shows how the roadmap for MHP disease management could be constructed. An animated version of this figure, illustrating the dynamics of this roadmap, can be found as Additional file 1.

#### Step 1. Monitoring the disease/pathogen and the hosts/environment

Following the discovery of a disease outbreak, priority must be conferred to descriptive studies and disease/pathogen monitoring, which should be both initiated as early as possible. It is the step 1 of our roadmap (Fig. 1).

By improving the quality and quantity of available data (on the disease/pathogen, the host-pathogen interactions, the host populations and the pathogen/environment interactions), descriptive studies improve the outcome of decisions that will be made regarding disease management. Descriptive studies should aim at defining the epidemiological role of each affected species and helping selecting the units relevant to disease management [18].

In case of a disease outbreak, monitoring also enables public health officers to adapt the disease management plan to any evolution of the epidemiological situation in a timely manner (i.e., adaptive management). For this purpose, monitoring should provide sufficient spatial resolution to “allow changes in disease occurrence to be identified and the impact of any intervention to be critically assessed” [18].

Besides disease/pathogen monitoring, monitoring of the host population(s) (population ecology and dynamics) and the interactions between the pathogen and the environment is essential to assess the potential ecological impacts of the management plan (impacts on the concerned wild animal population but also on the ecosystem it belongs to).

Overall, monitoring activities after a disease outbreak should include the collection of all possible data that can be gained from the implemented disease and population management activities, from the very beginning to the end and even beyond the outbreak. In our opinion, engaging in cost savings by minimizing data collection within the framework of an outbreak can result in higher costs if another event of the same type occurs again, as the existing gaps in knowledge may affect the prediction and mitigation plans for this new event.

Research has been progressing in fields that are relevant to wildlife health and wildlife population monitoring. Wildlife surveillance networks (event-based or targeted surveillance) [19] and diagnostic tools (e.g., high-speed sequencing, nucleic acid microarray chips) have improved and accelerated disease detection (thanks to higher test sensitivity, the availability of rapid and transportable tests, the collection of temperature-resistant sample matrices, and improved communication within and beyond national borders) and increased possibilities for field-testing, resulting in shorter delays between clinical suspicion and diagnostic confirmation. These improvements may increase the number of detected outbreaks and lead to a growing need for decisions to be made by competent authorities. More efficient early detection of diseases should also be associated with earlier management actions (when these are needed) and, in turn, result in more efficient disease control. The progress in statistical methods (e.g., for geographical and temporal analyses) ensures that increasing amounts of information are retrieved from disease events and improves the output of disease/pathogen monitoring (e.g., hindcasting techniques using early outbreak data to predict the trajectory of an epidemic prior to detection; [20]. Overall, there is a wide set of diagnostic, statistical and modelling methods which are increasingly performant and can be used to improve the quality and quantity of knowledge on wildlife population and disease/pathogen dynamics that can serve decision making.

#### Step 2. Evaluating the risk

Disease outbreaks raise a question that is difficult to answer: “is the sanitary risk acceptable for the exposed population(s), and is action needed to lower the disease risk to an acceptable level?” To answer this question, risk assessment is necessary prior to choosing a course of action. Risk assessment must be recognized as a compulsory step prior to disease management because decision makers need to balance the perception of a need for action with the actual current and future disease risk. In the OIE Terrestrial Animal Health Code, risk assessment is defined as “the evaluation of the likelihood and the biological and economic consequences of entry, establishment and spread of a hazard” [21]. This evaluation corresponds to step 2 of our proposed roadmap (Fig. 1).

Risk assessment is based on the collective, independent and potentially contradictory deliberations of experts, selected in the absence of conflicts of interest and based on their experience in the relevant fields of expertise (including wildlife when this unit is concerned). It aims to set qualitative or quantitative thresholds in terms of pathogen circulation within each affected unit by identifying a level of risk within this unit. That level of risk (which can be qualified as negligible, minor, moderate, high, or unknown) is estimated based on the current knowledge on transmission routes, host and pathogen ecology and contact structures. This risk assessment should be performed as early as possible, and it should take into consideration not only what is known but also what is not known. Therefore, the process of risk assessment also requires the characterization of sources of uncertainty, (i.e., the lack of data on specific elements of the outbreak), the expected inaccuracies (e.g., the limited sensitivity and/or specificity of diagnostic tests used for surveillance) and variations in the available data (e.g., natural biological variation). This is particularly important in the case of wildlife because the level of uncertainty is generally higher than for other units. Choosing courses of action without taking into account the uncertainties can have deleterious effects.

Risk assessment is not supposed to define management goals or suggest courses of action. It rather contributes to define an acceptable risk level from which a global management goal can be defined and subsequent management goals for each unit can be derived. However, while we do believe that risk assessment is essential before invasive measures (culling, vaccination, sterilisation) are envisaged, we consider that the emergency implementation of common sense measures to protect specific compartments or limit the expansion of an outbreak (i.e., non-invasive, short-term and consensual actions such as a reinforcement of biosecurity or hunting bans) should not be postponed until completion of risk assessment. Importantly, risk assessment should include evaluating the potential negative consequences of disease control measures.

Competent bodies exist to provide these assessments at the national level (e.g., the French Agency for Food, Environmental and Occupational Health and Safety ANSES in France, the Federal Agency for the Safety of the Food Chain AFSCA in Belgium, or the Bundesinstitut für Risikobewertung BfR in Germany) and at the European level (European Food Safety Agency, EFSA). Risk assessment performed by these independent agencies is a transparent process and yields clear results on the risk level and on the level of (un) certainty it is based upon. It is therefore essential to communicate the results of the risk assessment (which includes information on environmental issues linked to the origin and impact of diseases, and on potential cascades of management consequences such as a potential disequilibrium of the affected ecosystem) to all stakeholders, such as farmers, hunters, conservationists and other concerned land users within the span of an outbreak.

All the scientific information used to evaluate the risk should be presented and referenced in written reports enabling science-based communication and constituting a knowledge file for eventual management plans. A complete file should include up-to-date information on transmission routes, host and pathogen ecology, contact structures and expected impacts (economical, ecological, and sociological) of the disease.

#### Step 3. Determining the goals of disease management

Based on the results of the risk assessment, at first, an overall management goal should be set for the complete host-pathogen/disease system, which is step 3 of the roadmap we propose (Fig. 1). Once this overall disease management goal has been agreed upon, specific management goals have to be set for each epidemiological unit.

Wobeser’s classification [22] of management goals is generally used for the management of a transmissible disease. These possible goals are: prevention, control, eradication and “Laissez-faire” (do nothing). In the context of multi-host disease management, these four categories must be considered both for the complete disease system as well as for each single unit (appropriate goals may differ among units).

1. Prevention: this approach “includes all measures designed to exclude or prevent the introduction of a disease into an unaffected population”. Prevention can be appropriate for an unaffected country or for unaffected units (e.g., a pathogen circulating in wildlife that is not yet affecting captive animals or humans).
2. Control: this strategy “applies to activities designed to reduce the frequency of occurrence or the effects of an existing disease within an individual animal or a population to an acceptable or tolerable level, or to contain the spatial spread of infection.” Control implies the acceptance of a certain level of pathogen persistence in one or more units. Depending on the context, this approach can correspond either to long-term continuous action to limit or cap disease prevalence, or to short-term measures prior to the implementation of an eradication program.
3. Eradication: this approach “involves the total elimination of an existing disease.” Fifty years of experience have shown that eradicating disease or infection that implicates wildlife is a long-term issue. In fact, efforts to eradicate diseases such as TB, brucellosis or CSF are still ongoing in Europe. When the eradication of MHP was achieved (which remains an exception), this process took years, or in some cases, even decades [23].
4. “Laissez-faire” means to “refrain from harming”. This approach can be compared to the adage “*primum non nocere*” [first, do no harm] that guides the medical professions towards a prudent and proportionate response to curing a disease. Laissez-faire remains a baseline scenario in terms of the cost/efficiency relationship. Nevertheless, it requires targeted surveillance and communication to the exposed public, and such an approach is actually far from doing nothing. This management approach (rather than goal) must be considered (1) when disease mitigation is considered necessary but efficient management tools are not available/not possible, and (2) when a risk/reward analysis shows that it is the best choice. Disease outbreaks in wildlife can indeed slow down and spontaneously die out through self-regulation as was the case for *Brucella melitensis* infections in Alpine ibex (*Capra ibex*) in Gran Paradiso National park (Italy) [24]. Furthermore, it is possible that human intervention may not be efficient [25] or may even be counter-productive [26].

The choice of the general and specific management goals is based on the expected impacts of the disease on animals and humans, on the respective roles of each unit in the epidemiology of the disease of concern, on the level of circulation of the pathogen within and among the different units, and on the host/pathogen interactions. The four management goals listed above are generic and need to be more precisely defined in operational situations. Future efforts on the decision-making framework we present in step 4 of our roadmap (Fig. 1) should ultimately focus on setting numerical objectives (e.g., specific incidence or prevalence to attain acceptable pathogen circulation, deadline for the eradication of a pathogen, incidence of a pathogen under which doing nothing is a valid goal).

#### Step 4. Selecting effective and proportionate management actions: a decision-making framework

We have developed a decision-making framework for guiding competent authorities in their selection of management actions to achieve the general and unit-specific management goals defined in step 3. This decision-making framework is presented in Additional file 2 and its corresponding user’s guide is presented in Additional file 3. It aims at enabling managers to select the appropriate set of actions through a logical process and from a comprehensive checklist of possible procedures. In its current stage of development, it is a concept model consisting in an Excel® file comprising three worksheets. It is not a user ready decision-making framework (possible improvements of the model are presented in a dedicated paragraph).

To be effective, disease management actions have to sufficiently contribute to breaking the epidemiological links (direct and indirect) between the reservoir (i.e. the infected unit/population(s), which maintain the pathogen and act as a source of infection for other units/hosts) and the spill-over unit(s)/host(s) (i.e., the sensitive population(s) exposed to the reservoir) [25, 27]. The prevention, control or eradication of a disease implies an integrative used of all of the necessary management actions, based on each particular epidemiological scenario and its evolution.

Although additional aspects of MHP disease system may also dictate the final choice of disease management actions, the initial selection from the checklist of available actions will depend on the pathogen transmission pathways (e.g., disease management actions aiming at affecting pathogen survival in the environment are of little interest for directly transmitted diseases). We therefore chose to develop one worksheet per transmission pathway, proposing a checklist of suitable actions per pathway. Although there are only three main possible pathways (direct, indirect and vector-borne), their possible combinations result in seven transmission scenarios. To cover all the scenarios within three sheets, we proceeded as follows: the “direct transmission” worksheet presents only management actions relevant for direct transmission; the “indirect transmission” worksheet present management actions for both direct and indirect transmission; and the “vector-borne transmission” worksheet presents management actions for vector-borne, indirect and direct transmission. A simplified version of these worksheets is presented in Table 1.

**Table 1:** Simplified version of the decision making framework

Units	Feasibility	Cost	Efficiency	Action to be considered for management plan
Humans	Choose management goal for this unit: Prevention, Control, Eradication or Laissez-faire			
*Action 1*	Availability?	Economical?	Efficient alone?	Yes or No?
	Possibility?	Social?	Efficient combined with other options?	
	Capacity?	Environmental?	Inefficient?	
*… …*	…	…	…	…
*Action x*	…	…	…	…
Captive animals	Choose a management goal for this unit: Prevention, Control, Eradication or Laissez-faire			
*Action 1*	Availability?	Economical?	Efficient alone?	Yes or No?
	Possibility?	Social?	Efficient combined with other options?	
	Capacity?	Environmental?	Inefficient?	
*... …*	…	…	…	…
*Action x*	…	…	…	…
Environment	Choose a management goal for this unit: Prevention, Control, Eradication or Laissez-faire			
*Action 1*	Availability?	Economical?	Efficient alone?	Yes or No?
	Possibility?	Social?	Efficient combined with other options?	
	Capacity?	Environmental?	Inefficient?	
*......*	…	…	…	…
*Action x*	…	…	…	…
Wildlife	Choose a management goal for this unit: Prevention, Control, Eradication or Laissez-faire			
*Action 1*	Availability?	Economical?	Efficient alone?	Yes or No?
	Possibility?	Social?	Efficient combined with other options?	
	Capacity?	Environmental?	Inefficient?	
*… ..*	…	…	…	…
*Action x*	…	…	…	…

This simplified table illustrates how the process of choosing the relevant management actions should be followed. This table is a simplified version of one worksheet which corresponds to one type of disease transmission. For each relevant unit (humans, captive animals, environment and wildlife), each listed management action should be weighed against the following factors: feasibility, cost and efficiency for the pre-established management goal (eradication of the disease, control of the disease, prevention of the disease or laissez-faire). Following this process should leave the manager with a list of one or more management actions for each unit, which match the 3 criteria and should be considered for implementation

Once the relevant sheet has been selected, each management action should be weighed against three factors as follows: (1) feasibility, (2) cost, (3) efficiency.

Feasibility is the first question to address. Factors to take into account are the availability of the action (e.g., is a vaccine available, and as an appropriate formula?) and the capacity to attain operational objectives for this action (e.g., can a vaccination campaign cover a sufficient proportion of the population to prevent pathogen maintenance?). Some management actions require specific administrative authorisations which can delay or condition their implementation and render them unavailable as short-term actions (culling of protected species).

Cost comes as a second question. It is not only about material costs (financial costs including manpower) but also about the ecological “costs” or negative impacts on ecosystem health (e.g., what would be the impact on non-target species ingesting vaccinal baits?).

Expected efficiency is the third and final question to answer (e.g., can vaccination efficiently reduce the occurrence of a disease/pathogen to attain eradication within the unit?). The expected efficiency is conditioned by the type of action, by the host/pathogen interactions, by the host ecology and perhaps even more factors. It is important to remember that single actions may be inefficient and that it is often necessary to consider combining several measures.

The aim of this procedure is to build a disease management plan that includes both efficient and proportionate responses. The notion of proportionate management implies that the measures taken to manage a disease outbreak are not more costly than absolutely needed to decrease the pathogen circulation to an acceptable level (i.e., the impact of the action to be undertaken should not overreach its efficiency). Factors to consider when aiming at proportionate management include the expected efficacy of the measures for the target species, the social acceptability and feasibility of the measures, and the ecological cascade of expected consequences. The term “acceptable” refers to a risk assessment that accounts for all stakeholder points of view, which implies that compromises will be made because these points of views may be contradictory. The “proportionate response” notion is present in the EU Animal Health Regulation (Article 82), but its local implementation relies on the local authorities that face a disease outbreak.

The decision-making framework we present in this article is a preliminary attempt to integrate scientific knowledge on disease epidemiology, monitoring and control and on population dynamics into a simplified but complete and rational table. An improved version of this decision-making framework could be used by the competent authorities to select management actions for each of the affected units, by following the integrative approach promoted by the “One Health” concept. The output of this decision-making framework is quite straightforward and simple and can be used as a basis for presenting potential disease management measures to decision makers.

The first operational experiences with this decision-making framework are likely to reveal which improvements are needed. For the logical process that consists of a succession of conditional functions, the initial spreadsheet could be presented as a software that integrates more information for each step in the future. As an example, the SANTERO (risk-based Surveillance for ANimal HealTh in EuROpe) consortium developed a web tool serving as a surveillance design framework and another web tool to help designing the assessment methods for the chosen surveillance design [28].

Although it is important that such a tool is easy to use and that it presents simple, straightforward results, a certain level of complexity is inevitable, because it deals with MHP diseases, which are the most complex disease systems and are accordingly difficult to study, monitor and manage. Passing from a spreadsheet format to a web tool or software would enable its simplified use (i.e., by only displaying necessary fields) and broaden its possibilities, such as subdividing the units if several species are susceptible. Although the format of the decision-making framework could certainly be improved and more possibilities considered, we believe that the basic principles and parameters that have been used to prepare the current framework will continue to apply to future models.

The use of an agreed-upon decision-making framework should limit the potential for conflicts and ensure a multi-disciplinary approach enabling full transparency among disciplines and stakeholders. Furthermore, the decision-making framework as we designed it requests that the manager address the three crucial questions of disease management (feasibility, cost and efficiency). This approach also sets the criteria used to assess the efficiency of the management measures after their implementation.

None of the information and data available on a disease system can be directly transposed from the study results into the decision-making framework. It is also not possible for the results of the framework to be directly applied in the field. This tool remains a technical support to prioritize disease management actions but it is not sufficient to deliver a final management plan to public and animal health authorities. The output of this tool is, and should remain, open to interpretation by properly trained and competent personnel. This restriction highlights once more the importance of a transdisciplinary collaboration because the use of the tool requires competencies in human and animal health, ecology and wildlife management.

#### Step 5. Regularly assessing the disease management plan: dynamic management

The process we present for choosing disease management actions only allows designing an efficient and proportionate management plan in response to the initial situation. Management actions aim at engendering changes in the initial epidemiological situation and the roles played by the affected units. These actions can be fully or partially efficient, non-efficient or even counter-productive; they can also have lower or higher negative impacts than expected. Therefore, it is essential for a management plan to be adaptable according to the occurring disease system’s changes (dynamic management). It implies that the management plan includes the potential downgrading of the actions in case of a favourable situation, as well as exit strategies in case of a failure in the first set of measures. This highlights the importance of continuous disease/pathogen and population monitoring (as recommended in step 1 of the roadmap) to identify these changes, and ultimately it means that both management plan (step 4) and monitoring plan (step 1) loop back, at a pre-established deadline, to risk assessment (step 2), which is then performed on a wider base of knowledge (on the pathogen, the hosts, their interactions and their reactions to management actions) and maybe the availability of new control techniques, and finally integrates an assessment of the initial management and monitoring plans.

Theoretical ecological models continually improve our basic understanding of the impacts of ecological factors on the efficacy of disease/pathogen surveillance [29] and on wildlife population control [30]. In turn, the higher resolution of pathogen monitoring and growing knowledge of wildlife ecology and population dynamics can be used to improve the predictive power of operational models for specific disease systems. These models enable us to simulate and develop disease control strategies and policy before an outbreak occurs or during an outbreak, representing a valuable tool for dynamic management. These advances in ecological understanding and control strategy development offer increased support to decision makers. For example, in recent events such as the detection of brucellosis in Alpine ibex in the French Alps and the emergence of ASF in wild boar in Eastern Europe, both the ANSES and EFSA included predictive models in their assessments [31, 32] within reasonable delays following disease detection. These modelling possibilities should be more systematically explored when management actions are planned for wildlife, to test the expected efficiency and impacts of different management options.

### Towards harmonized transboundary disease management

The five-step process we propose was built upon the successes and failures of wildlife health management in past decades. It offers a rational support for disease management decisions and may promote harmonized procedures in Europe. Indeed, disease surveillance in wild animal populations is often not straightforward, and surveillance networks are not evenly developed across Europe [19]. Wild animals can move freely and unchecked across borders and can sometimes even migrate over long distances. Pathogens that circulate within wild animal populations are therefore more difficult to record than those that circulate in captive animal populations [33]. The responsibility for disease surveillance and management does not rely on a single European country or EU member state [34]. The efficient management of transboundary multi-host diseases cannot be achieved without a global and coordinated response, which should be based on a common decision frame. Management plans should cover the whole span of a disease system, regardless of state frontiers, and with the same methods and means applied on the whole area. For the EU more specifically, this coordination is all the more feasible because of a common concept of animal health and the existence of a common regulatory frame, as embodied by the recent update of the Animal Health Regulation.

## Conclusions

Wildlife health management is complex and has its own specificities that differentiate it from captive animal health management and more generally from public health. In the last 50 years, public and animal health authorities have treated sanitary threats from wildlife on a case-by-case basis. However, the recently adopted EU Animal Health Law creates a need for guidelines to achieve proportionate responses to disease outbreaks that include a wildlife host. The significant improvement of tools for diagnostics and data management provides an appropriate basis for moving forward. We propose to follow a systematized five-step process or roadmap as a way to recognize the specificities of wildlife and to integrate them into a wider approach (One Health). This process encompasses disease/pathogen and host population monitoring, disease risk assessment, setting unit-specific disease management goals, using a decision-making framework, and implementing dynamic management, with all of these steps to be systematically carried out.

## Additional files

Additional file 1: The management cycle for diseases implicating wildlife in a dynamic view. This video clip illustrates how the roadmap designed for the management of diseases implicating wildlife is to be used in a dynamic view. Step 1 -Descriptive studies and monitoring- is considered as ongoing for the whole duration of the outbreak (outer circle on the figure). Then the roadmap is constructed on 4 consecutive steps: (2) Risk assessment, (3) Management goals, (4) Management actions and (5) Assessment which are to be repeated as long as the outbreak persists (inner circle). (MP4 7438 kb)

Additional file 2: The decision-making framework. The decision-making framework is designed for the selection of management actions to achieve the general and unit-specific management goals. This framework is a spreadsheet consisting of five sheets. The first sheet is a general presentation which presents how the framework operates and invites the user to select the appropriate transmission pathways for the chosen diseases. To cover all the transmission scenarios, three sheets are then proposed: the “direct transmission” worksheet presents only management actions relevant for direct transmission; the “indirect transmission” worksheet present management actions for both direct and indirect transmission; and the “vector-borne transmission” worksheet presents management actions for vector-borne, indirect and direct transmission. For each relevant unit (humans, captive animals, environment and wildlife), each listed management action should be weighed against the following factors: feasibility, cost and efficiency for the pre-established management goal (eradication of the disease, control of the disease, prevention of the disease or laissez-faire). Following this process should leave the manager with a list of one or more management actions for each unit, which match the 3 criteria and should be considered for implementation. (XLS 148 kb)

Additional file 3: A user’s guide to the decision making framework. This document proposes a step-by-step user’s guide for operating the decision making framework presented as Additional file 2. (DOCX 17 kb)

## Data Availability

Not applicable.

## Abbreviations

AFSCA: Federal Agency for the Safety of the Food Chain in Belgium
ANSES: French Agency for Food, Environmental and Occupational Health and Safety
ASA association: Animal Société Aliment Association
ASF: African swine fever
BfR: Bundesinstitut für Risikobewertung
CSF: Classical swine fever
EFSA: European Food Safety Agency
EID: Emerging infectious disease
EU: European Union
HPAI: Highly pathogenic avian influenza
MHP: Multi-host pathogen
OIE: World Organisation of Animal Health
SANTERO: Risk-based Surveillance for ANimal HealTh in EuROpe
SRUC: Scotland’s Rural College
TAD: Transboundary animal diseases
TB: Tuberculosis

## References

[1] Gortazar C, Ferroglio E, Höfle U, Frölich K, Vicente J (2007). Disease shared between wildlife and livestock: a European perspective. Eur J Wildl Res.

[2] Naranjo V, Gortazar C, Vicente J, de la Fuente J (2008). Evidence of the role of European wild boar as a reservoir of mycobacterium tuberculosis complex. Vet Microbiol.

[3] Sikarskie JG, Miller RE (2008). Chapter 52 - tuberculosis in Michigan deer A2 - Fowler, Murray E. Zoo and wild animal medicine.

[4] Cleaveland S, Laurenson MK, Taylor LH (2001). Diseases of humans and their domestic mammals: pathogen characteristics, host range and the risk of emergence. Philos Trans R Soc Lond Ser B Biol Sci.

[5] Jones KE, Patel NG, Levy MA, Storeygard A, Balk D, Gittleman JL (2008). Global trends in emerging infectious diseases. Nature.

[6] Taylor LH, Latham SM, Woolhouse ME (2001). Risk factors for human disease emergence. Philos Trans R Soc Lond Ser B Biol Sci.

[7] Gortazar C, Reperant LA, Kuiken T, de la Fuente J, Boadella M, Martinez-Lopez B (2014). Crossing the interspecies barrier: opening the door to zoonotic pathogens. PLoS Pathog.

[8] Kock R (2014). Drivers of disease emergence and spread: is wildlife to blame?. Onderstepoort J Vet Res.

[9] EU: 2016/429 (2016). Regulation (EU) 2016/429 of the European Parliament and of the Council of 9 March 2016 on transmissible animal diseases and amending and repealing certain acts in the area of animal health (“Animal Health Law”).

[10] Haydon DT, Cleaveland S, Taylor LH, Laurenson MK (2002). Identifying reservoirs of infection: a conceptual and practical challenge. Emerg Infect Dis.

[11] Viana M, Mancy R, Biek R, Cleaveland S, Cross PC, Lloyd-Smith JO (2014). Assembling evidence for identifying reservoirs of infection. Trends Ecol Evol.

[12] Fehlner-Gardiner C, Nadin-Davis S, Armstrong J, Muldoon F, Bachmann P, Wandeler A (2008). Era vaccine-derived cases of rabies in wildlife and domestic animals in Ontario, Canada, 1989-2004. J Wildl Dis.

[13] Carter SP, Roy SS, Cowan DP, Massei G, Smith GC, Ji W, Delahay RJ (2009). Chapter 7: options for the control of disease 2: targeting hosts. Management of disease in wild mammals.

[14] Rossi S, Staubach C, Blome S, Guberti V, Thulke H-H, Vos A (2015). Controlling of CSFV in European wild boar using oral vaccination: a review. Front Microbiol.

[15] O’Brien DJ, Schmitt SM, Fitzgerald SD, Berry DE (2011). Management of bovine tuberculosis in Michigan wildlife: current status and near term prospects. Vet Microbiol.

[16] Mörner T, Obendorf DL, Artois M, Woodford MH (2002). Surveillance and monitoring of wildlife diseases. Rev Sci Tech Int Off Epizoot..

[17] Mick V, Le Carrou G, Corde Y, Game Y, Jay M, Garin-Bastuji B (2014). Brucella melitensis in France: persistence in wildlife and probable spillover from Alpine ibex to domestic animals. PLoS One.

[18] Gortazar C, Diez-Delgado I, Barasona JA, Vicente J, de la FJ, Boadella M (2015). The wild side of disease control at the wildlife-livestock-human interface: a review. Front Vet Sci.

[19] Kuiken T, Ryser-Degiorgis MP, Gavier-Widen D, Gortazar C (2011). Establishing a European network for wildlife health surveillance. Rev Sci Tech Int Off Epizoot.

[20] Rydevik G, Innocent GT, Marion G, Davidson RS, White PCL, Billinis C (2016). Using combined diagnostic test results to hindcast trends of infection from cross-sectional data. PLoS Comput Biol.

[21] OIE (2015). Terrestrial animal health code: Chapter 2.1. Import risk analysis.

[22] Wobeser G (2002). Disease management strategies for wildlife. Rev Sci Tech Int Off Epizoot.

[23] Roeder P, Mariner J, Kock R (2013). Rinderpest: the veterinary perspective on eradication. Philos Trans R Soc Lond Ser B Biol Sci.

[24] Ferroglio E, Tolari F, Bollo E, Bassano B (1998). Isolation of Brucella melitensis from alpine ibex. J Wildl Dis.

[25] MacDonald DW (1980). Rabies and wildlife. A biologist’s perspective.

[26] Gallagher J, Clifton-Hadley RS (2000). Tuberculosis in badgers; a review of the disease and its significance for other animals. Res Vet Sci.

[27] Begon M, Bennett M, Bowers RG, French NP, Hazel SM, Turner J (2002). A clarification of transmission terms in host-microparasite models: numbers, densities and areas. Epidemiol Infect.

[28] Risksur website. 2019. https://fp7-risksur.eu/.

[29] Walton L, Marion G, Davidson RS, White PCL, Smith LA, Gavier-Widen D (2016). The ecology of wildlife disease surveillance: demographic and prevalence fluctuations undermine surveillance. J Appl Ecol.

[30] Prentice JC, Marion G, White PCL, Davidson RS, Hutchings MR (2014). Demographic processes drive increases in wildlife disease following population reduction. PLoS One.

[31] ANSES (2015). Mesures de maîtrise de la brucellose chez les bouquetins du Bargy -Avis de l’Anses, rapport d’expertise collective.

[32] EFSA panel on Animal Health and Welfare (2015). African swine fever -Scientific opinion.

[33] Artois M, Delahay RJ, Guberti V, Cheeseman C (2001). Control of infectious diseases of wildlife in Europe. Vet J.

[34] Voyles J, Kilpatrick AM, Collins JP, Fisher MC, Frick WF, McCallum H (2015). Moving beyond too little, too late: managing emerging infectious diseases in wild populations requires international policy and partnerships. EcoHealth.

