# Supplementary material for: Multi-host disease management: the why and the how to include wildlife
Source: BMC Vet Res. 2019 Aug 14;15:295. doi: 10.1186/s12917-019-2030-6 (PMC6694651; doi:10.1186/s12917-019-2030-6)
Supplement: Supplementary file 3 — A user’s guide to the decision making framework. This document proposes a step-by-step user’s guide for operating the decision making framework presented as Additional file 2. (DOCX 17 kb) [file 12917_2019_2030_MOESM3_ESM.docx]

**Annex 2: General instructions for using the Decision making framework**

**Sheet 1:** instructions for use. Users are invited to follow these three steps:

1. Select the transmission route(s) of the pathogen (direct, indirect and/or vector-borne) and describe the details of the transmission route(s):
   1. direct transmission: bites, contact with blood, saliva and other secretions or excretions containing infectious organisms, venereal and food-borne or predation/scavenging
   2. indirect transmission via fomites: inhalation, ingestion, inoculation and contact
   3. vector transmission: air-borne vectors (culicids or mosquitoes, dipteral insects in general) and terrestrial vectors (primarily ticks and fleas)
   4. a combination of several transmission routes
2. Fill in the checklist of measures on the sheet corresponding to the transmission route(s) of the disease (see below). Even if there are multiple transmission routes, the users are invited to fill in only one sheet; the “direct transmission” sheet only presents management options for direct transmission, the “indirect transmission” sheet presents management options for both direct and indirect transmission, and the “vector-borne transmission” sheet presents management options for vector-borne, indirect and direct transmission. Therefore, all seven transmission scenarios are listed in the three sheets.

a. The direct transmission sheet should only be filled in for direct transmission pathways.

b. The indirect transmission sheet should be filled in if there are indirect transmission pathways with or without direct transmission pathways.

c. The vector-borne sheet should be filled in if vector-borne transmission pathways are present with or without other transmission pathways.

1. Read the summary of the options on the last sheet.

**Sheets 2, 3 and 4:** (sheet 2 for direct, sheet 3 for indirect and sheet 4 for vector-borne transmission) checklists of the options classified by epidemiological unit (humans, captive animals, environment and wildlife). On each sheet, column 1 indicates the management option (one option per line), and column 2 gives a description of the essence of the option. The units are ordered as follows: humans, captive animals, environment and wildlife. Within each unit, the management options are ordered from the priority ones at the top (less invasive) to the last-resort ones at the bottom (the most invasive, e.g., culling for domestic animals or wildlife). For each option (line), columns 3 to 9 are to be filled in by the user as follows:

1. Column 3: indicate the selected management goal (Prevention, Control, Eradication, or Laissez-faire, with one selection per unit)
2. Column 4 to 6: inform about the feasibility, cost and efficiency
3. Column 7: according to the information listed in columns 3 to 6, choose if the option should be considered or not as part of the management plan
4. Column 8: fill in this column with comments on measure implementation if necessary (possible difficulties, critical points to address)
5. Column 9: fill in the monitoring possibilities, because changes due to a certain measure must be detectable through monitoring schemes
6. Within each compartment, stop this process as soon as the sum of measures that is deemed relevant is sufficient to attain management objectives.

**Sheet 5:** a synthesis of measures to implement in reaction to the pathogen. On this last sheet, the users have nothing to fill in.

1. The first table recapitulates the route(s) of transmission by automatically copying the answers from the first table of sheet 1.
2. The second table is composed of 2 columns (Measures, To implement or not) and summarizes the primary measures to be implemented, with the answer “yes” or “no” being automatically filled in (using the conditional function “IF… THEN… ELSE”) by copying the answers from the column “To be considered or not” of the sheet corresponding to the route of transmission.
3. After filling in all the cells (for Feasibility, Cost, and Efficiency), the manager can then choose to consider the management option by selecting Yes/No in the “To be considered for management” cell. At the end of the process (all options have been studied and selected or left blank), the selected options are presented in a separate sheet, which provides an overview of the chosen options for all the suitable units.
